# Alveolar-capillary endocytosis and trafficking in acute lung injury and acute respiratory distress syndrome

**DOI:** 10.3389/fimmu.2024.1360370

**Published:** 2024-03-12

**Authors:** Vitalii Kryvenko, István Vadász

**Affiliations:** ^1^ Department of Internal Medicine, Justus Liebig University, Universities of Giessen and Marburg Lung Center (UGMLC), Member of the German Center for Lung Research (DZL), Giessen, Germany; ^2^ The Cardio-Pulmonary Institute (CPI), Giessen, Germany; ^3^ Institute for Lung Health (ILH), Giessen, Germany

**Keywords:** clathrin-mediated endocytosis, caveolae, endophilin, glycosylphosphatidyl inositol-anchored protein enriched early endosomal compartment pathway, macropinocytosis, phagocytosis, trafficking, acute respiratory distress syndrome

## Abstract

Acute respiratory distress syndrome (ARDS) is associated with high morbidity and mortality but lacks specific therapeutic options. Diverse endocytic processes play a key role in all phases of acute lung injury (ALI), including the initial insult, development of respiratory failure due to alveolar flooding, as a consequence of altered alveolar-capillary barrier function, as well as in the resolution or deleterious remodeling after injury. In particular, clathrin-, caveolae-, endophilin- and glycosylphosphatidyl inositol-anchored protein-mediated endocytosis, as well as, macropinocytosis and phagocytosis have been implicated in the setting of acute lung damage. This manuscript reviews our current understanding of these endocytic pathways and subsequent intracellular trafficking in various phases of ALI, and also aims to identify potential therapeutic targets for patients with ARDS.

## 1 Introduction

Endocytosis is a complex process involving formation of membrane vesicles that are involved in the transport of various molecules from the extracellular space or plasma membrane into the cytoplasm. The cargo usually contains transmembrane proteins and diverse extracellular ligands varying from small solutes to pathogens and even infected and apoptotic cells (1–4). Moreover, internalized molecules participate in a wide range of physiological processes, including nutrition, cell signaling, and cell adhesion (1, 2, 5). Generally, the endocytic process begins with the selection of cargo molecules from the extracellular environment, followed by recruitment of cargoes to the forming endocytic site. After selection, a budding vesicle is generated. This vesicle is connected to the plasma membrane through the involvement of assembling proteins. These proteins may include clathrin/caveolin or curvature sensing/stabilization proteins. After the formation of the endocytic invagination, the invagination neck is eventually constricted and cut to release the endocytic vesicle inside the cell. This process is known as vesicle scission (1, 6). The last step may be dynamin-dependent or independent. Currently, six major types of endocytosis have been identified, depending on the involvement of assembling proteins and the action of dynamin: 1) clathrin-coated pit-mediated endocytosis (CME; clathrin and dynamin dependent), 2) caveolae-mediated endocytosis, 3) fast endophilin-mediated endocytosis (FEME; a clathrin-independent but dynamin-dependent pathway for rapid ligand-driven endocytosis of specific membrane proteins), 4) clathrin-independent carrier (CLIC)/glycosylphosphatidyl inositol-anchored protein enriched early endocytic compartment (GEEC) endocytosis (clathrin and dynamin independent), 5) macropinocytosis and 6) phagocytosis (7). The process of endocytosis relies on various cellular components, including coat proteins, adaptors, regulatory factors, cytoskeletal proteins and molecules essential for intracellular trafficking (3). After internalization, the cargo undergoes intracellular trafficking ultimately leading to its recycling, transcytosis or degradation (2, 8, 9).

In patients with acute respiratory distress syndrome (ARDS), a devastating consequence of various pulmonary and extra-pulmonary insults, such as severe pneumonia, aspiration of gastric contents, sepsis, major trauma and shock, the alveolar-capillary barrier progressively fails and protein-rich edema floods the alveolar space (10). As opposed to physiological conditions, during which the lungs are responsible for adequate gas exchange through the intact air-blood barrier, in the setting of acute lung injury (ALI) a diffuse alveolar damage (DAD) is characteristic. According to histology, ALI/ARDS can be classified into three phases: acute/early or exudative, organizing or proliferative, and resolution or fibrotic phase (11). In the first phase, massive destruction of the alveolar-epithelial barrier is observed. This is accompanied by an increased inflammatory response, leakage of protein-rich fluid into the alveolar space and dysregulation of alveolar-fluid balance. Later, the resolving and/or organizing phases occur. These involve alveolar epithelial cell differentiation, alveolar-epithelial barrier regeneration with subsequent clearance of edematous fluid from the alveoli and/or fibroblast proliferation (10, 12, 13).

**Figure 1:**
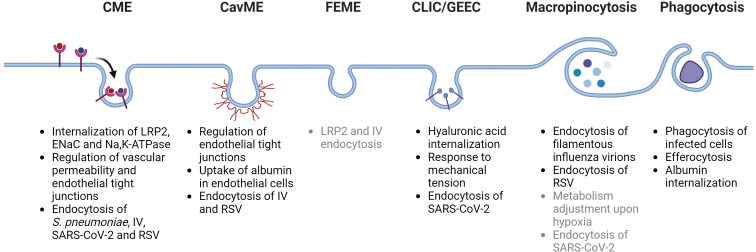
Schematic depiction of main endocytosis pathways and their potential roles in acute lung injury and acute respiratory distress syndrome. CME, clathrin-mediated endocytosis; CavME, caveolae-mediated endocytosis; FEME, fast endophilin-mediated endocytosis; CLIC/GEEC, clathrin-independent carriers/GPI-AP enriched early endosomal compartment; LRP2, low density lipoprotein receptor-related protein 2 (also known as megalin); ENaC, epithelial sodium channel; IV, influenza virus; RSV, respiratory syncytial virus; SARS-CoV-2, severe acute respiratory syndrome-related coronavirus. Potentially-related mechanisms are listed in gray color. Created with BioRender.com.

Endocytic processes are essential to maintain alveolar-capillary barrier function and play a key role in the initial injury of alveolar epithelial and endothelial cells as pathogens or the associated danger-molecules reach these compartments during ALI/ARDS (10, 13). Moreover, endocytosis is also a central regulator of processes that remove edema fluid and excess protein from the alveolar space, which are essential to restore normal alveolar fluid balance and thus, for patients with ARDS to survive (14, 15). Dysregulation of endocytic mechanisms during ALI and ARDS can contribute to more pronounced lung damage and ultimately to irreversible deleterious remodeling of the lung (16, 17). Currently, there is no specific pharmacological treatment for patients with ARDS. Thus, understanding the key mechanisms of endocytosis and their relevance in the context of ALI and ARDS (as depicted in **Figure 1**) may help to develop novel therapeutic targets and strategies for these patients.

## 2 Endocytic pathways in the setting of ALI/ARDS

### 2.1 Clathrin-coated pit-mediated endocytosis

Clathrin-coated pit-mediated endocytosis (CME) is considered to be the major and best-studied pathway for cargo internalization. It has been shown that in 95% of cases the earliest detectable endocytic vesicles originate from clathrin-coated pits (18). CME is the best understood endocytic pathway and is often referred to as receptor-mediated endocytosis. The first step in CME is the formation of a clathrin coat. In addition to clathrin, this coat consists of over 60 clathrin-adaptor and scaffold proteins. The clathrin adaptor proteins, the heterotetrameric AP2 complex and several monomeric proteins, including the clathrin assembly lymphoid myeloid leukemia protein (CALM) family and epsins, bind to lipids in the plasma membrane and to cargo molecules (1). The function of scaffold proteins, such as clathrin, epidermal growth factor receptor substrate 15 (EPS15), epidermal growth factor receptor substrate 15-like 1 (EPS15R) and intersectins is to interact with clathrin adaptors and cluster the coat components together (1). Subsequently, the formation of the clathrin coat leads to actin filament polymerization, forming the actin module. This process requires, among others, members of the Wiskott-Aldrich syndrome protein (WASP) family and actin related proteins (such as Arp2/3) (19, 20). After forming the invagination neck, constriction and scission occur. These processes are mediated by Bin-Amphiphysin-Rvs (BAR) domain proteins, such as endophilins and amphiphysins, and are followed by uncoating (1, 21).

The formation of protein-rich alveolar edema due to failure of the alveolar-epithelial barrier is a hallmark of ALI/ARDS (10, 22). Under physiological conditions, the concentration of albumin in the alveolar lining fluid is much lower than in plasma (22). Early reports suggested that albumin was endocytosed in epithelial cells by albondin (also known as gp60). It has been shown that albumin and gp60 co-localize in epithelial cells, suggesting that gp60 is involved in albumin endocytosis. Of note, the use of the caveolae-disrupting agent filipin was able to attenuate albumin internalization (23), raising the question whether albumin is endocytosed by clathrin- or caveolae-dependent mechanisms. A series of subsequent studies suggested that albumin is predominantly internalized by clathrin-dependent mechanisms in epithelial cells (24–27). Direct comparison between alveolar type I (ATI)-like and type II (ATII) alveolar epithelial cells showed higher uptake of albumin under cell culture conditions in ATI-like cells (26). In another study, isolated ATI and ATII cells derived from precision-cut lung slices (PCLS) from mice showed comparable uptake rates of albumin (25). To assess which molecules drive CME-mediated albumin uptake in the lung, recent research focused on the significance of the megalin/clathrin axis in albumin endocytosis in lung epithelial cells (28–30). These studies established that megalin, a multi-ligand receptor for various proteins in the kidney, is an albumin receptor in the alveolar epithelium (28). Importantly, in the setting of ALI activity of transforming growth factor (TGF)-β is elevated that mediates activation of the glycogen synthase kinase 3 (GSK3)-β, leading to internalization of megalin from the plasma membrane. This results in a decrease in albumin endocytosis and thus protein and edema clearance from the alveolar compartment of injured lungs (30). Moreover, in addition to GSK3-β activation, TGF-β reduces megalin surface expression through activation of matrix metalloproteinases (MMPs) that drive shedding of the albumin receptor in alveolar epithelial cells (29). In contrast to epithelial cells, albumin uptake in endothelial cells appears to be primarily caveolin dependent; however, it has also been proposed that endothelial albumin uptake may also occur through a caveolae-independent pathway involving CME and subsequent accumulation of albumin in endosomes (31).

In addition, the resolution of alveolar edema depends on active sodium transport processes through the coordinated action of the apically localized epithelial sodium channel (ENaC) and the basolateral Na,K-ATPase (15, 32). This creates a Na^+^ gradient that drives the movement of excess water out of the alveolar space, resulting in alveolar fluid reabsorption (12, 33). It has been shown that expression of both molecules is dramatically decreased during various settings of respiratory failure and ALI/ARDS (14, 34–37). Of note, it has been demonstrated that CME plays a key role in the endocytosis of both ENaC and Na,K-ATPase. For example, after ubiquitination driving endocytosis, ENaC has been found to be present in clathrin-coated vesicles in epithelial cells. Moreover, epsin-1 has been identified as an accessory protein, further suggesting involvement of CME in ENaC endocytosis (38). Similarly, endocytosis of the Na,K-ATPase appears to be a clathrin-mediated mechanism dependent on phosphorylation of Ser18 and binding of the CME adaptor protein AP2 to the Na,K-ATPase α_1_-subunit (39, 40).

Furthermore, alterations in the expression levels of proteins involved in CME may influence susceptibility to infections and development of lung edema. For example, increased expression of neural WASP has been linked to elevated vascular permeability and formation of pulmonary edema during *Pseudomonas aeruginosa* infection (41). Conversely, downregulation of WASP has been shown to improve survival rates and prevent pulmonary edema formation in a model of bleomycin-induced ALI (42). Indeed, bacterial and viral pathogens, or the combination of those in case of e.g. secondary bacterial infections in patients who were initially infected with respiratory viruses (43, 44), are the most common causes of ARDS (10). CME has been identified as a crucial factor in the internalization of these pathogens, primarily viruses, with subsequent ALI. Regarding bacteria, recent studies proposed that the internalization of *S. pneumoniae*, the most common pathogen causing pneumonia, involves several endocytosis pathways. It has been demonstrated that the internalization of *S. pneumoniae* is facilitated via both clathrin- and caveolae-mediated processes, as a significant inhibition of *S. pneumoniae* uptake was observed with chemical inhibitors of these pathways, including chlorpromazine and nystatin (45).

Several respiratory viruses have been implicated in the pathogenesis of virus-induced ALI/ARDS. Perhaps best studied among those include influenza virus (IV), severe acute respiratory syndrome-related coronavirus (SARS-CoV), SARS-CoV-2 and respiratory syncytial virus (RSV) (46, 47). The endocytic vesicles provide a significant advantage to viruses by enabling their movement from the cell periphery to the perinuclear region of the host cell, thereby facilitating their entry into the nucleus (3). Moreover, the decreased pH in the endosomal environment and altered redox state enable viruses to recognize their location. In addition, once endocytosed, viruses are able to conceal their presence at the plasma membrane, thus delaying their detection by the immune system (3).

Regarding influenza, it is well documented that after IV binds to the host receptor sialic acid at the plasma membrane, clathrin-coated pit formation is initiated, indicating that CME plays a major role in cellular uptake of the virus (48). Additionally, it has been shown that epsin-1, a protein that interacts with clathrin, is involved in the CME of IV in epithelial cells, in a calcium-dependent manner (49, 50). Of note, a specific receptor for influenza endocytosis has not yet been identified. However, a recent study has shown that IV endocytosis requires transferrin receptor 1 (TfR1). Genetic and chemical gain-of-function and loss-of-function experiments have confirmed the involvement of TfR1 in IV entry. It is interesting to note that even non-glycosylated TfR1 mutants function as an entry receptor for IV, despite the crucial role of sialic acid in IV endocytosis (51).

CME also plays a crucial role in internalization of the spike glycoprotein of SARS-CoV through the angiotensin-converting enzyme 2 (ACE2) receptor (52). It was shown that either treatment with the CME inhibitor chlorpromazine or knockdown of the clathrin heavy chain protein significantly inhibited internalization of SARS-CoV in HepG2 and fibroblast-like COS7 cells (53). Furthermore, the use of dynasore and pitstop 2, which are known inhibitors of CME, along with knockdown of the clathrin heavy chain protein, was found to reduce internalization of the SARS-CoV-2 spike protein, strongly suggesting a role for CME in internalization of these coronaviruses (54). Finally, recent studies show that RSV utilizes CME and the subsequent early endosomal compartment during infection of human epithelial cells (55, 56). Treatment with optate, a compound that alters airway surface and intracellular pH, was able to inhibit RSV infection in primary human airway epithelial cells *in vitro*, suggesting a pH-dependent entry in the cell and potential involvement of the CME machinery (57). Interestingly, after internalization RSV activates Rab5a, a protein localized in early endosomes, and suppresses interferon regulatory factor 1 (IRF1)-dependent lambda interferon production, thereby reducing the antiviral defense activity of airway epithelial cells (58).

Regarding the endothelial compartment of the distal lung, vascular endothelial (VE)-cadherin is an important adhesion molecule involved in the maintenance of vascular alveolar-capillary barrier function. Various reports suggest that VE-cadherin undergoes internalization in clathrin-coated vesicles, indicating predominantly CME (59, 60). Considerable research has been conducted on the trafficking of VE-cadherin. One of the key molecules regulating VE-cadherin plasma membrane expression is p120-catenin (p120) (61, 62). VE-cadherin’s cytoplasmic tail contains a juxtamembrane domain (JMD) that directly binds to p120 (63, 64). Studies have shown that p120 binding to VE-cadherin’s JMD stabilizes the protein’s plasma membrane levels by preventing its clathrin-dependent endocytosis and subsequent degradation (65). Depleting p120 from human dermal microvascular endothelial cells resulted in decreased expression of VE-cadherin and overall decreased barrier function (66). In contrast, overexpressing p120 in human endothelial cells increased VE-cadherin levels and inhibited its degradation (67). It has been shown that p120 stabilizes both endothelial VE-cadherin and epithelial E-cadherin. In mouse lung epithelial cells and in an *in vivo* setting, overexpression of p120 inhibited E-cadherin endocytosis and occludin degradation maintaining both adherence and tight junctions in ventilator-induced lung injury (68). Moreover, in a bleomycin-induced model of ALI, p120 expression was increased in lung fibrotic loci *in vivo*, similarly to primary lung fibroblasts exposed to bleomycin. In addition, downregulation of p120 expression resulted in a decrease in pulmonary fibrosis in mice exposed to bleomycin (69).

However, not only p120 regulates internalization of VE-cadherin. Studies have shown that Rab4 and Rab9 regulate the cell surface abundance of VE-cadherin in the pulmonary endothelium, thereby regulating vascular permeability (70). Moreover, expression of VE-cadherin at the plasma membrane and its recycling have been found to be dependent on Rab11 and Rab11a. Silencing Rab11 and Rab11a was effective in preventing VE-cadherin recycling and markedly increased vascular permeability in an ALI model secondary to polymicrobial sepsis (71). Rab5 is a crucial regulator of microvascular endothelial integrity in the lung. The treatment with lipopolysaccharide (LPS) was found to significantly increase the expression of Rab5, which was associated with the internalization of VE-cadherin. In human lung microvascular endothelial cells, inhibition of Rab5 enhances barrier function by reducing permeability (72).

### 2.2 Caveolae-mediated endocytosis

Caveolae-mediated endocytosis (CavME), in contrast to other endocytic pathways, exhibits high cell- and tissue-specificity (7). Caveolae possess a unique morphology, representing a bulb- or omega-shaped pit connected to the plasma membrane by a small neck (73). Caveolae are predominantly located in epithelial, endothelial and smooth muscle cells, as wells as fibroblasts and alveolar macrophages in the lung (74, 75). Caveolae are formed by caveolin and cavin proteins, where cavins are peripheral membrane proteins that associate with caveolae under steady-state conditions (7). It has been demonstrated that downregulation of caveolin-1 (Cav-1), caveolin-3 (Cav-3) or cavin1 expression can block the formation of caveolae (7). In addition to caveolae formation, caveolins are integral membrane proteins that associate with several intracellular organelles including endosomes, the Golgi complex and lipid droplets (7).

Numerous studies have investigated the role of caveolae-associated proteins in ALI. Cav-1 has a well-known role in the maintenance of epithelial and endothelial tight junctions (76, 77). The knockdown of Cav-1 in the lung endothelium of mice *in vivo* caused a significant increase in pulmonary vascular permeability due to the disruption of endothelial junctions (78). It is noteworthy that LPS modulates the Cav-1/nuclear factor ‘kappa-light-chain-enhancer’ of activated B-cell (NF-κB) axis *in vivo*. Downregulation of Cav-1 in mice in LPS-induced ALI reduced inflammation by decreasing expression levels of CD3 and the adhesion family member F4/80, activated autophagy by inhibiting AKT/mTOR, and promoted AMP-activated protein kinase signaling, thereby improving lung morphology and function (79).

As mentioned above, megalin is centrally involved in albumin uptake in epithelial cells by CME. Conversely, albumin endocytosis and transcytosis in endothelial cells heavily rely on CavME. Studies have demonstrated that gold-conjugated albumin was not endocytosed in blood vessels of Cav-1-deficient lung endothelial cells. Subsequent experiments with radio-labeled albumin confirmed these results and showed little or no uptake in the aortic segments of Cav-1 null mice compared to wild-type animals (80).

Regarding the role of CavME in uptake of respiratory viruses, recent data suggest that in addition to CME, caveolae-mediated IV entry may also occur. For example, lower IV titers were observed in Madin-Darby canine kidney (MDCK) cells infected with knockdown or dominant-negative Cav-1 mutant strains. In addition, Cav-1 was found to modulate IV replication, presumably through the interaction between influenza protein M2 and Cav-1 (81). However, another study suggested that the presence of Cav-1 reduced IV replication in mouse fibroblasts (82). In the case of coronaviruses, it has been shown that Cav-1 interacts with orf3a, the largest unique open reading frame in SARS-CoV (83). However, whether CavME plays an active role in SARS-CoV-2 infections is not known. In contrast, it appears that in human epithelial HeLa cells caveolae is a specialized membrane environment for RSV virus assembly and that Cav-1 interacts directly with RSV G-protein (84). Further experiments in cultured and primary alveolar epithelial and endothelial cells as well as PCLS should be performed to identify the role of CavME in the endocytosis of respiratory viruses. For example, knock-out of Cav-1, Cav-3 or cavin1 with subsequent infection and determination of virus titers in these settings may help to dissect the role of CavME in virus infections.

### 2.3 Fast endophilin-mediated endocytosis

Fast endophilin-mediated endocytosis (FEME) was recently identified as an important pathway for rapid endocytosis of specific transmembrane receptors in a clathrin-independent but dynamin-dependent manner (7). Up to date, two families of endophilins have been identified: endophilin A1, A2, A3 and endophilin B1, B2 (85). Members of the endophilin A family are predominantly localized at the plasma membrane and have been shown to be involved in the process of endocytosis through interaction with dynamin, synaptojanin and amphiphysin I (85, 86). In contrast, endophilin B is mostly intracellularly localized and has been shown to be involved in mitochondrial morphogenesis, autophagy processes and apoptosis (87, 88). However, recent publications have revealed that endophilin B is additionally involved in endocytic trafficking (89–91). In particular, depletion of endophilin B impairs endosomal trafficking *in vitro*, as demonstrated by suppressed endosome acidification, EGFR degradation and autophagic flux (89). Upon binding of the cargo to the specific receptors, the activation of FEME is extremely rapid (92), which requires endophilin pre-enrichment in the plasma membrane as a FEME carrier (7, 92). The primary cargoes for the FEME pathway involve G-protein-coupled receptors, such as β_1_-adrenergic, dopaminergic, acetylcholine receptors, and the receptor tyrosine kinases, EGFR, HGFR, VEGFR, PDGFR, NGFR and IGF1R, as well as interleukin-2 receptor (7, 86, 92). Of note, β-adrenergic receptors (also expressed in the bronchial system) have been identified as relying exclusively on FEME for cellular entry and are therefore considered a classical cargo utilizing this form of endocytosis (7).

There is limited knowledge on the direct role of FEME in ALI/ARDS. It has been shown that TGF-β, one of the key mediators involved in the early injury and the late fibroproliferative phase of ARDS (93, 94), activates GSK3-β in alveolar epithelial cells thus downregulating megalin expression on the plasma membrane in a dynamin-dependent manner (30). Notably, GSK3-β has also been identified as a kinase involved in the direct regulation of FEME via phosphorylation of dynamin and amphiphysin (95). This raises an interesting question as to whether the downregulation of megalin in ALI is also controlled by FEME. Moreover, it has previously been shown that two FEME cargoes, EGFR and VEGFR, are centrally involved in the pathogenesis of ALI/ARDS (96, 97).

As to the potential involvement of FEME in respiratory viral infections in the setting of ALI/ARDS, although the role of the endophilin A family in lung function or viral endocytosis is not thought to be relevant, endophilin B may play an important role IV infections. In particular, it has been demonstrated that in mouse embryonic fibroblasts endophilin B2 deficiency impairs the entry of IV RNA in the nucleus and thus replication of the virus (89). In line with these findings, another study showed that mice lacking endophilin B2 had a decrease in IV load in the lung and had better recovery after infection with the H1N1 PR8 strain (98).

### 2.4 Clathrin-independent carriers/glycosylphosphatidyl inositol-anchored protein enriched early endosomal compartment pathway

The fourth endocytic pathway operates independently of clathrin or dynamin and is termed clathrin-independent carriers/glycosylphosphatidyl inositol-anchored protein (GPI-AP) enriched early endosomal compartment (CLIC/GEEC) pathway (7, 86). Unlike FEME that relies on activation by ligand-receptor interactions, CLIC/GEEC spontaneously occurs in the cell. This pathway is responsible for the internalization of certain surface proteins, including hyaluronic acid receptor (CD44) and in some cases, the uptake of fluid and membrane by cells. The CLIC/GEEC pathway is regulated by ADP-ribosylation factor 1 (ARF1), Golgi-specific brefeldin A resistant factor 1 (GBF1), the actin related protein complex Arp2/3 and small GTPase cell division control protein 42 (Cdc42) (86, 99). In addition, recent studies have shown that the CLIC/GEEC pathway is partially dependent on the extracellular excretion of galectin-3, which interacts with lipids, proteins, CD44, and integrins (100).

Hyaluronic acid (HA) is a crucial component of the extracellular matrix (ECM) and is ubiquitously expressed throughout the lung (101). During ALI, a significant destruction of the ECM is observed. Studies have demonstrated that levels of HA are elevated in both bleomycin-induced ALI and in patients with ARDS (102, 103). The HA receptor CD44, internalization of which is mediated by the CLIC/GEEC pathway, plays a prominent role in the resolution of ALI in both *in vitro* and *in vivo* models. Research has shown that CD44 has a protective effect in hyperoxia- and LPS-induced ALI and that CD44 deficiency results in a significant influx of inflammatory cells and increased chemokine expression in the broncho-alveolar lavage fluid (BALF) (104, 105). This can be explained by the preserved internalization of HA by CD44. However, it is not yet clear to what extent the CLIC/GEEC mechanisms is involved in the internalization of CD44 during ALI, and thus, further investigations addressing this point are needed. To address these questions, binding and uptake studies with genetic or chemical inhibition of key proteins involved in the CLIC/GEEC pathway, such as galectin-3, ARF1, GBF1, should be performed. In addition, the use of *in vivo* ALI models and patient samples, such as BALF and subsequent analysis of the transcriptome, proteome and phosphokinome of alveolar epithelial and endothelial cells will expand the understanding of the involvement of the CLIC/GEEC signaling pathway in ALI./ARDS.

This pathway has also been proposed in the context *S. pneumoniae* infections, as these bacteria may use, apart from CME, a dynamin-independent pathway, as CLIC/GEEC for cellular entry (106). It is noteworthy that the CLIC/GEEC pathway may also contribute to endocytosis of SARS-CoV-2 under specific circumstances. A recent study demonstrated that the receptor-binding domain (RBD) of the SARS-CoV-2 spike protein is internalized through the CLIC/GEEC pathway (107). However, this observation was made in human gastric adenocarcinoma cells, which do not express ACE2. In contrast, inhibition of Cdc42 in HEK293T cells expressing ACE2 had no effect on viral titer following infection with SARS-CoV-2 spike-carrying pseudoviruses (108).

The lungs undergo constant stretching during inspiration and expiration, which is largely aggravated in the setting of ARDS due to the lung injury and the mechanical ventilation that this patient group very frequently requires. A recent study demonstrated that Cdc42-driven actin remodeling and the consequent activation of mitogen-activated protein kinases are crucial for alveolar regeneration in response to mechanical tension in the lung (109). Moreover, exposing epithelial and mesenchymal cells to stretching led to the activation of the CLIC/GEEC pathway by altering the expression of GBF1 on the plasma membrane through vinculin, a protein that functions as a mechanotransducer (110). If not resolved, deleterious remodeling including fibrotic processes occurs at the late stages of ALI/ARDS, where integrins play a key role. Integrins are glycoprotein receptors that are important for cell adhesion and tissue integrity. Their involvement in lung repair and the development of pulmonary fibrosis, asthma, emphysema, and acute lung injury is well established (111, 112). Of note, Cdc42, a central regulator of the CLIC/GEEC pathway, is one of the downstream targets of integrins (112). Interestingly, loss of Cdc42 function in alveolar stem cells prevents regeneration of alveoli in elderly mice and also post pneumonectomy leading to increased mechanical tension, activated TGF-β signaling and progressive lung fibrosis (113). These results prompt the question of whether dysregulation of the CLIC/GEEC signaling pathway is implicated during mechanical ventilation and in the resolution phase of ALI in patients with ARDS. Future experiments should investigate the effects of Cdc42 knockout in alveolar epithelial and endothelial cells. Additionally, it would be interesting to determine if stimulation with bleomycin or LPS activates the CLIC/GEEC pathway and expression of Cdc42.

### 2.5 Macropinocytosis

During macropinocytosis actin-driven extensions of plasma membrane layers are formed that non-selectively incorporate extracellular fluid and solutes into large intracellular vesicles (macropinosomes) (114). Once the macropinosome has closed, it transforms to enter the early endosomal compartment (7, 115). This endocytic pathway is crucial in the absorption of extracellular substances, nutrition, and antigen presentation (115). Dextran represents the classical cargo for macropinocytosis. However, dextran with low molecular mass (<10 kDa) can act as a liquid phase marker and is incorporated into all forming endocytic vesicles. This therefore makes dextran with low molecular weight unsuitable for characterizing the macropinocytic pathway (7).

There is limited information about the role of macropinocytosis in ALI/ARDS. Most research has focused on the role of this type of endocytosis in cancer settings and its role in tumor invasion and metastasis (116). Studies have shown that hypoxia can activate the HIF-1 pathway and EH domain-containing protein 2 (EHD2), leading to the initiation of macropinocytosis in tumor cells and their increased growth (117). It is currently unclear whether hypoxia, a common condition during ARDS, contributes to changes in cell metabolism by activating macropinocytosis in lung cells. Interestingly, research indicates that EGFR triggers macropinocytosis, resulting in increased nutrient absorption at the tip of lung branches. Consequently, this process contributes to the morphogenesis of multicellular lung branches (118). However, it is not known if this mechanism is important during the resolution phase of ALI.

Moreover, current findings indicate that the endocytosis of IV may also occur, apart from CME, through macropinocytosis (119). Interestingly, a recent publication reports that influenza virus reduces albumin uptake and megalin expression in alveolar epithelial cells through MMP-driven shedding of the receptor, both in cultured alveolar epithelial cells and murine PCLS (120). This decline in the protein clearance under viral infection could have significant implications for outcomes of patients with ARDS. It has been revealed that both Rab5 and Rab7 are important for IV entry (121). Rab11 has been found to be a key factor in the influenza virus lifecycle, playing a role in both viral genome assembly and the subsequent release of influenza virions (122). Furthermore, it was demonstrated that viral influenza RNA particles directly bind Rab11 and compete with Rab11 family interacting proteins (FIPs) in human epithelial cells, promoting changes in protein and lipid homeostasis during infection (123). Studies have demonstrated that, contrary to spheroidal influenza viruses that mainly use CME, filamentous influenza viruses primarily utilize macropinocytosis as their entry mechanism (124). This is noteworthy given the frequent observation of filamentous influenza virions in human clinical infections (125). Influenza virions have been observed to enter cells as complete filaments within macropinosomes before being transported to the acidic late-endosomal compartment. Interestingly, low pH causes fragmentation of the influenza virus, enhancing membrane fusion between the viral and endosomal components (124). In addition, SARS viruses have been found to use macropinocytosis (126, 127). Basigin (CD147), one of the extracellular membrane receptors responsible for macropinocytosis of viruses in epithelial and endothelial cells (3, 128), has been found to interact with SARS-CoV-2 RBD domain in the human bronchial cell line BEAS-2B. Additionally, the treatment with CD147-neutralising antibodies was able to inhibit SARS-CoV-2 replication in human bronchial epithelial cells and fibroblasts (129). However, these findings were not validated in Calu-3 and Vero cells, which have high ACE2 receptor expression (130, 131). Finally, recent studies in human epithelial cells (HeLa and A549) as well as in human bronchial epithelial cells have demonstrated that RSV virus can also be taken up by micropinocytosis (132). Once attached to the cell surface, RSV induces rearrangement of actin via activation of Cdc42, and is then rapidly internalized by Rab5-positive macropinosomes (132).

### 2.6 Phagocytosis

Phagocytosis is a process by which professional phagocytes (neutrophils, monocytes, and macrophages) and non-professional phagocytes take up specific cargoes. It eliminates dead cells, cellular debris, and pathogenic microorganisms by the innate immune system (133). This endocytic process is triggered by the binding of particles to specific surface proteins such as scavenger receptors (133). Actin reorganization is crucial for extending the membrane and forming phagosomes. It is interesting to note that molecules that orchestrate this process, including Cdc42, the small GTPases Rac and RhoA, WASP and Arp2/3 are also key players during other endocytic processes, such as CME and the CLIC/GEEC pathway (7).

Regarding bacteria causing respiratory infections, phagocytosis has been shown to play an important role as an entry pathway for *L. pneumophila* and *M. tuberculosis* in macrophages (4, 134). In the context of ALI, phagocytosis has been demonstrated to play a central role in the apoptosis of virus-infected cells. For example, neutrophils and macrophages have been implicated in the phagocytosis of cells infected with IV (135). Furthermore, aging has been shown to impair the phagocytosis of alveolar macrophages and increase influenza mortality (136).

As discussed above, epithelial cells are capable of clearing excess alveolar proteins, which is crucial in resolving alveolar edema in patients with ARDS. As opposed to epithelial and endothelial cells, macrophages use phagocytosis as a mechanism for albumin clearance. Several reports have demonstrated that macrophages actively take up albumin in various cell culture models and in murine PCLS (25, 137, 138).

During ALI, there is significant death of epithelial and endothelial cells. Activated neutrophils have an extended lifespan, but they eventually undergo apoptosis (139). Phagocytosis of apoptotic cells is termed efferocytosis. It has been shown, that efferocytosis plays a critical role in the resolution of inflammation in the setting of ALI (139, 140). Of note, a recent clinical study demonstrated that patients with ARDS exhibit impaired alveolar macrophage efferocytosis (140). In murine models of ALI, the administration of efferocytosis activators, such as efferocytic opsonin milk fat globule epidermal growth factor-VIII (MFG-E8) or resolvin E1, has been associated with a decrease in cytokine influx, attenuation of lung inflammation, and increased survival (141–143). The receptor CD36, previously shown to be active in FEME and albumin endocytosis, plays a key role in modulating efferocytosis in the bleomycin-induced model of ALI (144). The downregulation of CD36 expression was associated with a delayed clearance of apoptotic alveolar cells and the progression of pulmonary fibrosis (144, 145). These findings make CD36 a promising candidate for therapeutic targeting in the setting of ALI.

## 3 Limitations and opportunities

Many questions remain to be answered despite decades of intense research in the field of endocytosis. As our models and methodologies become more sophisticated, we increasingly realize the complexity of these endocytic pathways, with often overlapping molecular players. Previously, the study of endocytic processes heavily relied on chemical inhibitors and silencing/overexpression of key proteins. However, more recent data suggest that chemical inhibitors are not as specific as previously believed. For instance, dynasore, which is often used as a CME inhibitor also interferes with FEME and influences macropinocytosis and phagocytosis (7). Furthermore, typical inhibitors of macropinocytosis, such as cytocholasin D or amiloride, have off-target effects on the CME, FEME and CLIC/GEEC pathways (86). Also, recent studies demonstrate that altering key components of one endocytic pathway can impair others. For example, overexpression of certain components of CavME, such as cavin-1, cavin-3, Cav-1 and Cav-3, results in inhibition of the CLIC/GEEC pathway (146). In fact, Cavin-1 is now known to function as an important regulator of Cdc42, a key element of the CLIC/GEEC pathway (146). Moreover, Cav-1 interacts with the EGFR receptor (147), which is a known FEME endocytosis cargo. Furthermore, specific conditions may inversely alter different endocytic process. For example, cigarette smoke appears to increase susceptibility to IV infection in lung epithelial cells by downregulating CME but upregulating CavME (148).

A limitation of pulmonary endocytosis research is the use of cellular lines. Much of the data on endocytic signaling pathways has been generated using non-pulmonary and non-primary settings, including HEK293T, HeLA and MDCK cell lines. Therefore, it is necessary to confirm some of the obtained data in primary lung epithelial and endothelial cells using *in vitro* and more complex *ex vivo* and *in vivo* systems. Furthermore, it is important to realize that certain cell lines that are often used in pulmonary research have a consistent state of activation or deactivation of various endocytic pathways. For example, in lung adenocarcinoma cells that have mutated Ras, such as the KRAS mutation, macropinocytosis was found to be a major route of amino acid delivery (149). Furthermore, studies have demonstrated that RAC-driven macropinocytosis of extracellular proteins is an adaptive metabolic pathway in non-small cell lung cancer cell lines when glucose is depleted (150). Thus, using such cell lines when studying endocytic and trafficking processes might be misleading (7).

In addition, a challenge arises in studying the binding and uptake of cargo in different endocytic systems. Several techniques including confocal, photon and super-resolution microscopy, fluorescent activated cell sorting (FACS) and application of pH-sensitive dyes are currently employed to distinguish binding, uptake of compounds, to investigate endocytic uptake from endocytic envelope formation to cytoskeletal rearrangement and endosome formation (151, 152). However, relying solely on fluorescent staining approaches may result in misleading conclusions. For instance, caveolin colocalization is regarded as a marker of CavME. However, Cav-1 overexpression leads to increased association with other endocytic pathways and endocytic compartments, resulting in off-target effects and outcomes (7). In addition, it is important to maintain the specific conditions required for the endocytic experiment. Using different temperature protocols for cargo internalization and uptake may interfere with endocytic pathways. CLIC/GEEC endocytosis has been found to recover at a slower rate from low temperatures than CME (7, 86).

In addition, the use of fluorescence microscopy to analyze cargo internalization is not sufficient, particularly for non-protein cargoes such as DNA, RNA or viruses. The main problem is the diffraction limit of light microscopy, which is above the size of the virus particles (153). Thus, recently various super-resolution fluorescence microscopy approaches, such as total internal reflection fluorescence (TIRF), stimulated emission depletion (STED) and single-molecule localization microscopy (SMLM) microscopy have been developed. These methods allow analysis of early steps in cargo internalization, such as binding to the plasma membrane, formation of the clathrin coat and interaction with actin filaments (151, 153). The development of single-cell sequencing approaches has made it possible to analyze the transcriptome of individual cells involved in endocytosis. However, spatial mapping of genes or internalized cargoes such as viruses is often limited. A new technology, Single-Cell Resolution IN Situ Hybridization On Tissues (SCRINSHOT), has been developed to address this issue, enabling amplification of viral RNA and host mRNA in a spatio-temporal manner (154). SCRINSHOT facilitates single-cell RNA mapping and could provide insights into the early stages of viral endocytosis prior to viral protein synthesis.

Various endocytic pathways have been demonstrated to be involved in the pathogenesis and sequel of ALI and ARDS. Endocytic processes are key during the initial insult, including viral and bacterial infections and their spread in the lung, as well as in altered alveolar-capillary barrier function and impaired resolution of injury. Development of new methodologies, the use of complex primary culture systems, such as PCLS, as well as single cell and spatial transcriptomics may provide answers to important questions about the role of endocytosis in the different phases of ALI and may ultimately identify new therapeutic targets for patients with ARDS.

## Author contributions

VK: Writing – review & editing, Writing – original draft. IV: Writing – review & editing, Writing – original draft.
